# A Cyclic-di-GMP signalling network regulates biofilm formation and surface associated motility of *Acinetobacter baumannii* 17978

**DOI:** 10.1038/s41598-020-58522-5

**Published:** 2020-02-06

**Authors:** Irfan Ahmad, Evelina Nygren, Fizza Khalid, Si Lhyam Myint, Bernt Eric Uhlin

**Affiliations:** 1 The Laboratory for Molecular Infection Medicine Sweden (MIMS) and the Department of Molecular Biology, Umea University, Umea, Sweden; 2 Institute of Biomedical and Allied Health Sciences, University of Health Sciences, Lahore, Pakistan

**Keywords:** Bacterial physiology, Bacteriology, Biofilms

## Abstract

*Acinetobacter baumannii* has emerged as an increasing multidrug-resistant threat in hospitals and a common opportunistic nosocomial pathogen worldwide. However, molecular details of the pathogenesis and physiology of this bacterium largely remain to be elucidated. Here we identify and characterize the c-di-GMP signalling network and assess its role in biofilm formation and surface associated motility. Bioinformatic analysis revealed eleven candidate genes for c-di-GMP metabolizing proteins (GGDEF/EAL domain proteins) in the genome of *A. baumannii* strain 17978. Enzymatic activity of the encoded proteins was assessed by molecular cloning and expression in the model organisms *Salmonella typhimurium* and *Vibrio cholerae*. Ten of the eleven GGDEF/EAL proteins altered the rdar morphotype of *S. typhimurium* and the rugose morphotype of *V. cholerae*. The over expression of three GGDEF proteins exerted a pronounced effect on colony formation of *A. baumannii* on Congo Red agar plates. Distinct panels of GGDEF/EAL proteins were found to alter biofilm formation and surface associated motility of *A. baumannii* upon over expression. The GGDEF protein A1S_3296 appeared as a major diguanylate cyclase regulating macro-colony formation, biofilm formation and the surface associated motility. AIS_3296 promotes Csu pili mediated biofilm formation. We conclude that a functional c-di-GMP signalling network in *A. baumannii* regulates biofilm formation and surface associated motility of this increasingly important opportunistic bacterial pathogen.

## Introduction

In recent years, *Acinetobacter baumannii* has emerged as one of the most problematic common opportunistic nosocomial pathogens worldwide. It is included in the group of bacterial pathogens termed as ESKAPE (*Enterococcus faecium*, *Staphylococcus aureus*, *Klebsiella pneumoniae*, *Acinetobacter baumannii*, *Pseudomonas aeruginosa* and *Enterobacter*spp.)^1–3^. Bacteria from this group efficiently escape the effect of many antimicrobial drugs and the WHO declared that *A. baumannii* is one of the most serious and antibiotic resistant ESKAPE organism^4,5^.

*A. baumannii* has not only gained resistance to antibiotics but also often has capability to resist disinfectants. Such resistant capabilities facilitate survival and colonization of *A. baumannii* in diverse environmental niches including hospital settings. It has an ability to colonize on both biotic and abiotic surfaces and can survive for long time under desiccation^3,6,7^. Biofilm formation of *A. baumannii is* one of the determinants of virulence and environmental persistence. *A. baumannii* is classified as a non-motile organism because of the lack of flagella^8^. However, many of the strains have ability to move on a semi solid surface in a manner termed as surface associated motility^9^. Also, type IV pili-dependent twitching motility has been reported for *A. baumannii*^10^.

Its notorious presence in hospital settings and increased mortality rate due to *A. baumannii* infections in intensive care units highlight the need to explore in depth underlying mechanisms behind its success as emerging pathogen. The bacterial second messenger rsignalling molecule c-di-GMP, originally discovered as an allosteric activator of cellulose synthase BcsA in *Gluconacetobacter xylinus*, has now been widely accepted as a key regulator of several bacterial traits including adaptation to harsh environments^11,12^. A wide range of bacterial characteristics are modulated by c-di-GMP signalling including transition from biofilm to motility, acute to chronic virulence characteristics, mutualism to commensalism and cell division to cell differentiation^13,14^. It has been shown that administration of c-di-GMP prior to infection may protect mice from *A. baumannii* lung infection^15^.

C-di-GMP is synthesized by diguanylate cyclase activity of GGDEF domain containing proteins. The key function of an active GGDEF domain is to catalyze the condensation of two GTP molecules to synthesize a c-di-GMP molecule with the release of pyrophosphate. The condensation reaction takes place at the active site upon the dimerization of two monomers of GGDEF domain protein^16^. Based on homology of entire GGDEF domains in combination with conservation of catalytic and substrate binding residues, the GGDEF domains can be differentiated into class I, class II and ClassIII^17^.

On the other hand, c-di-GMP is degraded into two GMP molecules by the phosphodiesterase activity of EAL domain containing proteins^13^. The intermediate product of EAL phosphodiesterase activity is the dinucleotide 5′-pGpG that is hydrolysed into two GMP molecules. Divalent cations Mg^2+^ or Mn^2+^ promote, while Ca^2+^ and Zn^2+^ efficiently inhibit, the enzymatic activity of EAL domain proteins. The entire EAL signature motif of active proteins consists of amino acids required for catalytic activity, binding of divalent cations and substrate binding. A flexible loop consisting of (β/α)_8_ barrels, known as “loop 6”, mediates dimerization of two monomers and controls substrate and cation binding. Based on conservation of residues required for enzymatic activity, substrate binding, metal binding and loop 6, the EAL domain proteins can be differentiated into three classes. Class 1 EAL proteins are enzymatically active; class 2 EAL domains are potentially active whereas class 3 EAL domain proteins are catalytically inactive proteins^18,19^. GGDEF/EAL proteins are often associated with amino terminal sensory domains. Of which the most important is PAS/PAC, Per (periodic clock protein), Arnt (Ah receptor nuclear translocator protein), Sim (single‐minded protein) domain proteins. The PAS domain can bind a diverse range of small-molecule metabolites. PAS ligand binding triggers a cellular signalling response through the C terminal domain with the capacity to respond to secondary physical or chemical signals such as gas molecules, redox potential, or photons^20^.

Apparent redundancy in GGDEF/EAL domain proteins in individual genomes is a hallmark of potential c-di-GMP signalling^21^. Usually GGDEF-EAL domains are linked to N terminal sensory domains and become activated upon ligand binding to the sensory domains. We considered that the putative genetic network for production and turnover of c-di-GMP in *A. baumannii* remained to be identified. Here we present the genetic and functional characterization of GGDEF/EAL proteins of *Acinetobacter baumannii* 17978.

## Results

### GGEDF/EAL proteins in the proteome of *Acinetobacter baumannii* 17978

**Figure 1:**
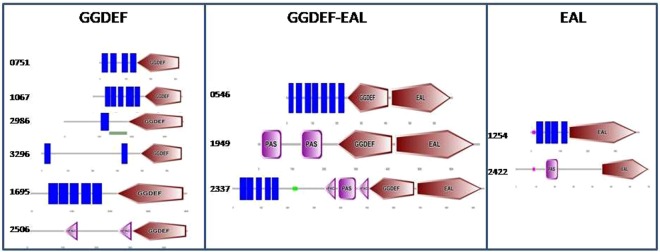
Predicted GGDEF/EAL proteins in *A. baumannii* 17978. Domain architecture of GGDEF/EAL proteins created by SMART protein analysis tool highlights the trans-membrane domains (Blue bars), PAS domains (Pink Box) and PAC domains (Pink arrowheads) in N terminal of GGDEF/EAL proteins.

**Figure 2:**
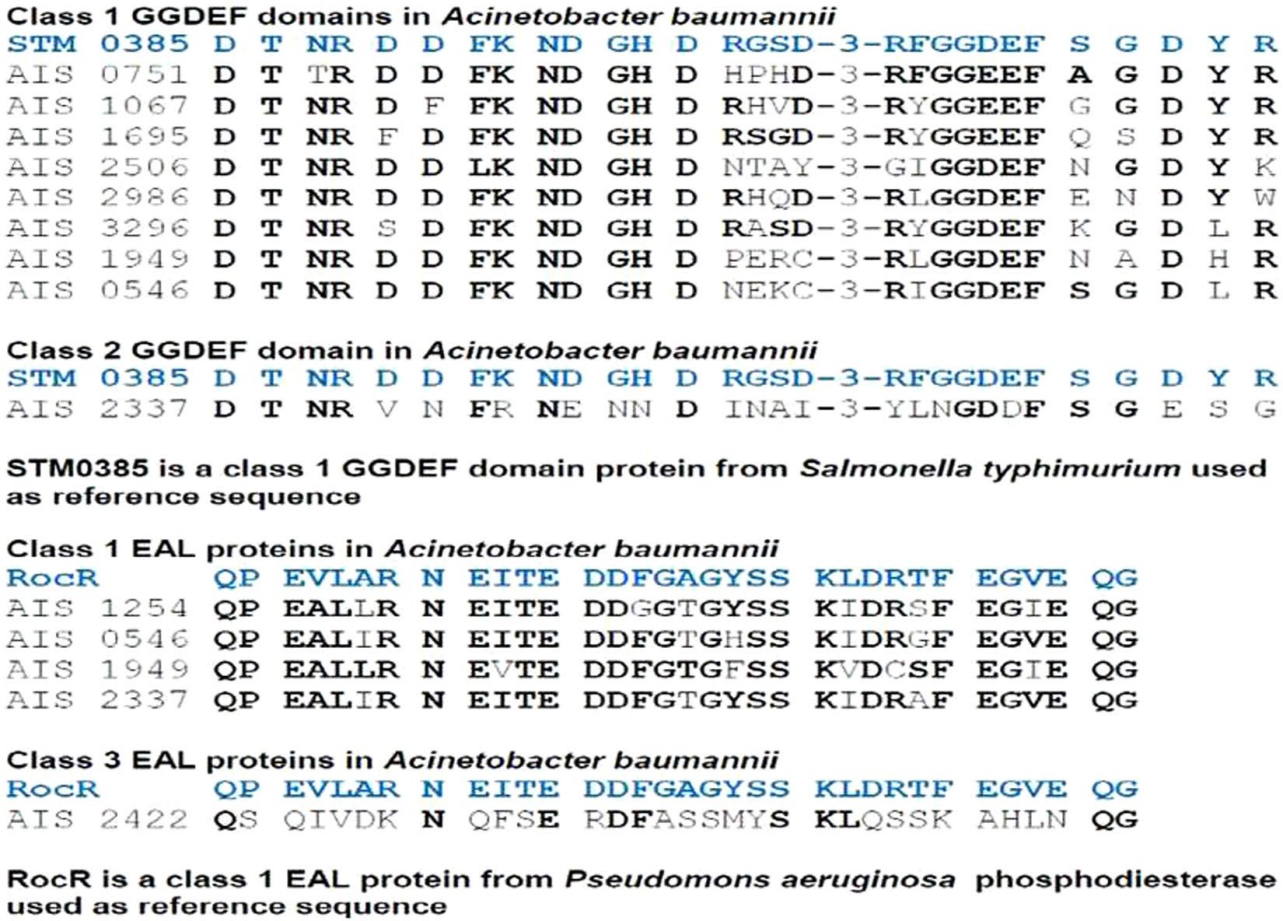
Conserved domain signature alignment and classification of predicted GGDEF/EAL proteins in *A. baumannii* 17978. The amino acid sequence of the GGDEF domain signatures of STM 0385 (AdrA), a diguanylate cyclase from *S. typhimurium* and RocR a phosphodiesterase from *P. aeruginosa* were used as reference sequences to align GGDEF/EAL proteins of *A. baumannii*. The conserved residues of GGDEF/EAL signature that perfectly aligned with the reference sequence are shown in bold face.

Blast searches in protein database (pfam) for GGDEF/EAL domains revealed the presence of eleven genes encoding for predicted GGDEF/EAL domain proteins in the genome of *Acinetobacter baumannii* 17978. Six proteins from genes located in the loci A1S_0546, A1S_0751, A1S_1067, A1S_1695, A1S_2986 and A1S_3296 contain GGDEF domains. Two proteins from genes located in the loci A1S_1254 and A1S_2422 contain EAL domains whereas three proteins from genes located in the loci A1S_0546, A1S_1949 and A1S_2337 contain both GGDEF and EAL domains. SMART analysis of the protein sequences indicated the presence of N terminal PAS domains in GGDEF-EAL proteins A1S_1949, A1S_2337 and EAL protein A1S_2422. The PAS domain of A1S_2337 was found to be sandwiched between two PAC domains. Two PAC domains also exist in the N terminal part of GGDEF protein A1S_2506. Eight of eleven proteins contain one or multiple transmembrane domains. Three proteins A1S_2422, A1S_2506 and A1S_1949 are lack of trans membrane domain and therefore, predicted to be cytoplasmic proteins (Fig. 1). Multiple sequence alignment of GGDEF proteins with a diguanylate cyclase AdrA from *Salmonella typhimurium* suggested that residues essentially required for diguanylate cyclase activity exist in eight of nine GGDEF domains of *A. baumannii*. Therefore, eight GGDEF domain proteins are predicted to be active diguanylate cyclases and belong to class 1 GGDEF domain proteins. The GGDEF domain of A1S_2337 however, harbors some deviations from a typical GGDEF domain (Fig. 2) and therefore, is classified as a class 2 GGDEF domain protein according to proposed classification scheme^21^. The multiple sequence alignment of EAL proteins of *A. baumannii* along with RocR, a phosphodiesterase from *Pseudomonas aeruginosa* shows that four EAL proteins contain residues required for phosphodiesterase activity and therefore were predicted to be phosphodiesterases. However, the EAL domain of A1S_2422 is deviating at multiple residues from a typical EAL domain. The substrate binding residues also deviated from that of the typical EAL domain protein RocR and therefore, A1S_2422 is proposed to be classified as a class 3 EAL domain protein according to defined criteria^22^ (Fig. 2).

### GGDEF/EAL domain proteins of *A. baumannii* 17978 exhibit enzymatic activity

**Figure 3:**
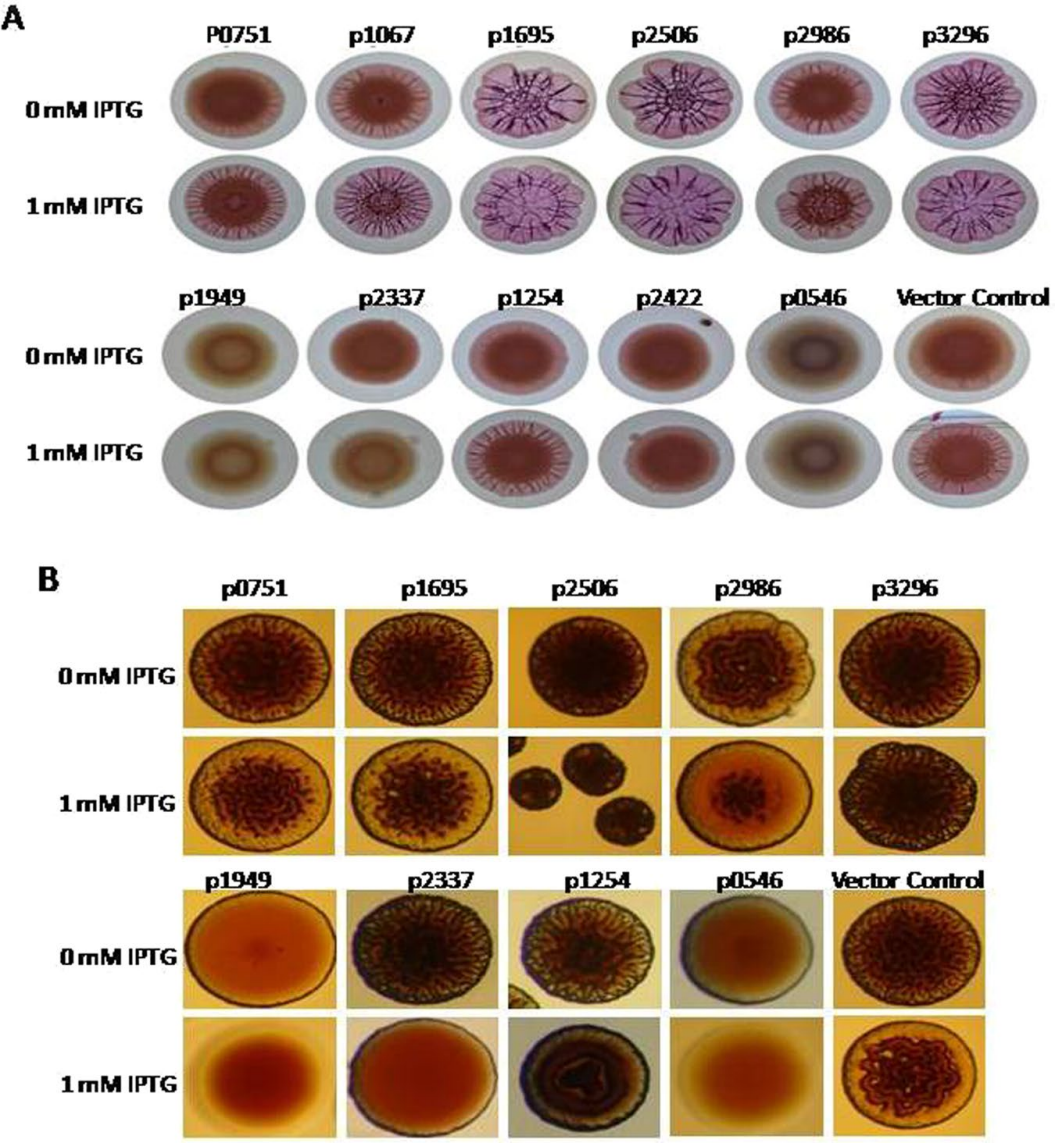
Assessment of the enzymatic activity GGDEF/EAL domain proteins of *A. baumannii* 17978 by phenotypic testsing in model organisms. (**A**) Rdar morphotype formation of *S. typhimurium* SR11 after 72 hours. (**B**) Rugose morphotype formation of *V. cholera* C6706*luxO*^*c*^after 48 hours. Derivatives of the two model strains were assessed upon the expression of individual GGDEF/EAL domain proteins of *A. baumannii* from the plasmid pMMB67EH on congo red agar plates.

In order to assess the enzymatic activity of GGDEF/EAL domain proteins of *A. baumannii*, the rdar morphotype of *Salmonella typhimurium* SR-11 and rugose morphotype of *Vibrio cholerae* C6706*luxO*^*c*^ were used as model systems. The rdar stands for rough, dry and red colony morphotype of *S. typhimurium* appeared upon the growth in petri plates supplemented with Congo red dye. The extracellular cellulose and amyloid curli fimbriae are two major extra cellular ingredients of rdar morphotype that bind Congo red dye^23^. It is well established fact that rdar and rugose morphotypes are promoted by c-di-GMP in respective organisms. The genes for GGDEF/EAL proteins were cloned into the broad host range plasmid pMBB67EH. The engineered constructs containing genes for GGDEF/EAL proteins were introduced by transformation into *S. typhimurium SR11* to assess any effect on rdar morphotype formation and into *V. cholerae* C6706*luxO*^*c*^ to assess the effect on rugose morphotype formation. The expression of GGDEF protein A1S_3296 through plasmid pMMB67EH was confirmed in *S. typhimurium* by Western blotting (Fig. S1). The rdar morphotype formation assay revealed that expression of six GGDEF domain proteins (A1S_751, A1S_1067, A1S_1695_A1S2506, A1S_2986 and A1S_3296) promoted the rdar morphotype formation of *S. typhimurium* SR11when compared to vector control (Fig. 3A). This finding is consistent with the predictions that these six proteins belong to class 1 and have a GGDEF domain that make them active as diguanylate cyclases. Interestingly, the extent to which rdar morphotype formation was displayed varied from protein to protein. A1S_1695, A1S_2506 and A1S_3296 exhibited stronger effect on rdar morphotype whereas A1S_1067, A1S_2986 and A1S_0751 exhibit a milder effect on the promotion of rdar morphotype. However, the GGDEF-EAL proteins A1S_1949, A1S_0546 and A1S_2337 suppressed the rdar morphotype formation. This finding suggests that the functionality as an active phosphodiesterase of C-terminally located EAL domains could be more pronounced in these proteins. No obvious effect of EAL proteins A1S_1254 and A1S_2422 on rdar morphotype formation was observed. The A1S_2422 protein would belong to class 3 EAL proteins and therefore phosphodiesterase activity is not expected. However, the lack of apparent enzymatic activity in the class 1 EAL protein A1S_1254 was unexpected and unusual.

The rugose colony formation of *V. cholera* C6706*luxO*^*c*^displayed an enhancement of rugose appearance by five GGDEF proteins, A1S_0751, A1S_1695, A1S_2506, A1S_2986 and A1S_3296, when compared to vector control. This finding is consistent with the observed enhancement of rdar morphotype by these proteins in *S. typhimurium*. Notably, the plasmid expressing one protein of class 1, the GGDEF protein A1S_1067, could not be introduced into *V.cholerae* strain C6706*luxO*^*c*^despite multiple transformation attempts. Consistent with results from tests monitoring the effect on rdar morphotype, GGDEF-EAL proteins A1S_0546, A1S_1949 and A1S_2337 suppressed the rugose morphotype formation (Fig. 3B). This finding suggests that GGDEF-EAL proteins A1S_0546, A1S_1949 and A1S_2337 function merely as phosphodiesteases. Notably, A1S_1254 also suppressed the rugose morphotype of *V. cholerae* but did not alter the rdar morphotype of *S. typhimurium* (Fig. 3A,B).

### GGDEF/EAL proteins regulate macro colony formation of *A. baumannii* 17978

**Figure 4:**
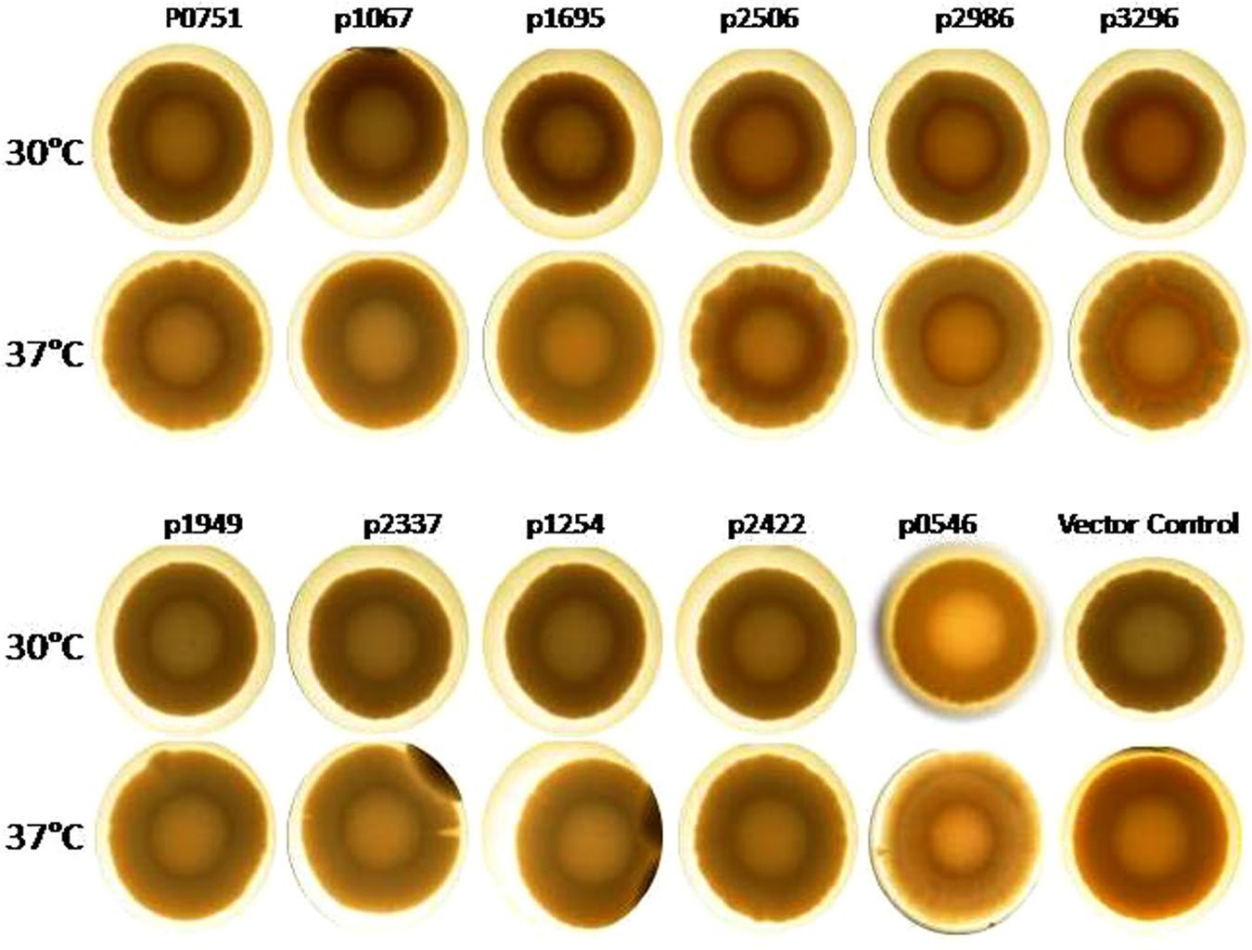
Development of macro colony formation in *A. baumannii*17978. (**A**) Stereo macroscopic images of single colonies of *A. baumannii* upon the expression of individual GGDEF/EAL domain proteins from the plasmid pMMB67EH. Growth was done on congo red agar plates supplemented with 1 mM IPTG and 100 μg/ml carbenicillin. The diguanylate cyclases A1S_2506 and A1S_3296 mediated expression of reddish matrix component at 30 °C and 37 °C after 48 hours.

After establishing phenotypically that several GGDEF/EAL proteins of *A. baumannii* indeed are active enzymes, we decided to investigate the effect of over-production of these proteins on macro colony formation of *A. baumannii*. Macro colony formation assay revealed that wild type *A. baumannii* 17978 forms smooth and white colonies on congo red agar plates at 30 °C and 37 °C (Fig. 4). The macro colony formation by *A. baumannii* expressing individual GGDEF/EAL domain proteins from the plasmid pMMB67EH was investigated on congo red agar plates supplemented with 100 µg/ml carbenicillin and 1 mM IPTG at 30 °C and 37 °C. Two of the predicted diguanylate cyclases, A1S_2506 and A1S_3296,were found to mediate the expression of a reddish matrix component in the middle of colonies at both 30 °C and 37 °C (Fig. 4) suggesting that these proteins may stimulate the production of extra cellular matrix components. Furthermore, upon over-production of the diguanylate cyclases A1S_0751, A1S_2506 and A1S_3296, the margin of colonies became uneven when compared to the wild type (Fig. 4).The rest of the GGDEF/EAL proteins seemed to be less effective in affecting the regulation of macro colony formation.

### Inverse regulation of biofilm formation by GGDEF protein A1S_3296 and GGDEF- EAL protein A1S_1949 in *A. baumannii* 17978

**Figure 5:**
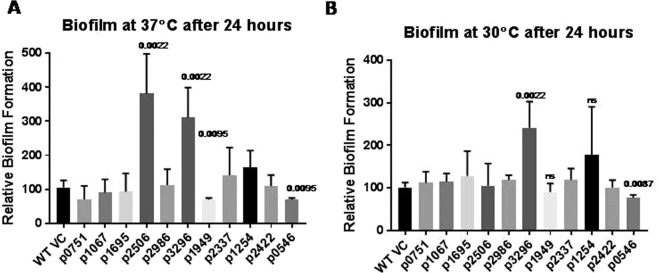
Biofilm formation assay of *A. baumannii* 17978 upon the expression of individual GGDEF/EAL domain proteins from the plasmid pMMB67EH. Biofilm formation assays were carried out at 37 °C (**A**) and 30 °C (**B**) as described in materials and methods with bacterial strains carrying the indicated plasmid clones. Error bars represent mean ± SD values of 6 replicates of three independent experiments*. P* values are shown on the top of columns with statistical significant alterations as compared to WT VC (Wild type vector control) were calculated by student paired *t* test using Graph Pad Prism software.

**Figure 6:**
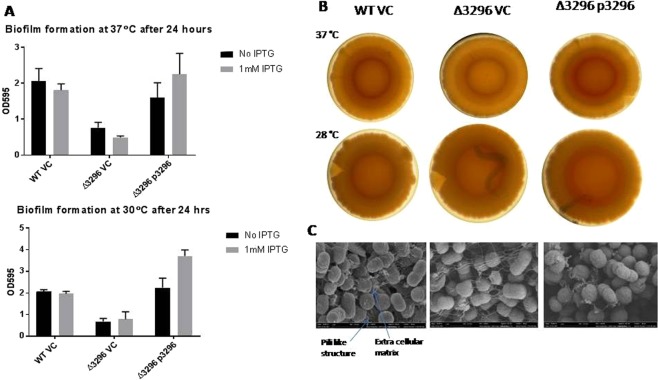
Analysis of biofilm formation by *A. baumannii* and of bacterial surface alterations upon deletion of the A1S_3296 coding sequence. (**A**) Biofilm formation assays were carried out at 37 °C and 30 °C as described in materials and methods with bacterial strains carrying the indicated plasmid clones. (**B**) Congo red binding monitored at the centre of colonies after growth at 37 °C and 28 °C. (**C**) Scanning electron microscopic images of colonies to reveal presence of extra cellular matrix component(s).

Next, we investigated the effect of over production of individual GGDEF/EAL proteins on biofilm formation of *A. baumannii* at 30 °C and 37 °C. Biofilm formation capability of *A. baumannii* was significantly enhanced upon the over-production of GGDEF proteins A1S_2506 and A1S_3296 whereas it was significantly reduced upon the over-production of GGDEF-EAL protein A1S_1949 at 37 °C. The rest of the GGDEF-EAL proteins did not exert statistically significant effects on biofilm formation at 30 °C or 37 °C. Interestingly, the effect of A1S_3296 was temperature independent whereas A1S_2506 was found to be effective only at 37 °C (Fig. 5A,B). These findings are consistent with the apparent stimulation of the extracellular matrix component by A1S_2506 and *A1S_3296* observed during macro colony formation (Fig. 4). The over-production of GGDEF-EAL protein *A1S_1949* (an apparent Phosphodiesterase) on the other hand, inhibited biofilm formation when compared to the vector control at 37 °C (Fig. 5A). Among all GGDEF/EAL proteins, A1S_3296 was found to be most effective in stimulation of biofilm formation. Therefore, a deletion mutant of *A. baumannii 17978* for the chromosomal gene encoding *A1S_3296* was created. Consistent with the high level of biofilm formation from over-production of A1S_3296, the deficiency in *A1S_3296* led to reduced biofilm formation when compared to wild type *A. baumannii* containing vector control (Fig. 6). Trans-complementation, i.e. the expression of A1S_3296 from the plasmid, restored the biofilm formation defect of the *A1S_3296* mutant strain at both 30 °C and 37 °C (Fig. 6A). Moreover, macro colony formation assay on Congo red agar plates revealed that congo red binding by the *A1S_3296* mutant strain was reduced when compared to wild type (Fig. 6B). However, such a difference was not visible at 30 °C. The expression of A1S_3296 from pMMB67EH promoted congo red binding in case of the A1S_3296 mutant of *A. baumannii* at both temperatures. In order to visualize extra cellular matrices produced by *A. baumannii* during macro colony formation, scanning electron microscopy of macro colonies was performed. SEM imaging revealed that *A. baumannii* grown as macro colony produced extra cellular pili as well as additional extracellular matrix components (Fig. 6C). Interestingly, deletion of the gene for protein A1S_3296 led to an increase in appearance of pili like structures and to a decrease in presence of extra cellular matrix components. The expression of A1S_3296 from the plasmid suppressed formation of pili like structures whereas it enhanced the secretion of extracellular matrix component(s) in case of the A1S_3296 mutant (Fig. 6C). These findings suggest multiple roles of A1S_3296 and c-di-GMP signalling in the regulation of biofilm formation in *A. baumannii 17978*.

### GGDEF/EAL domain proteins regulate surface associated motility of *A. baumannii* 17978

Up-regulation of biofilm formation and inhibition of motility is a fundamental function of c-di-GMP signalling in many bacteria. After the evaluation of the GGDEF/EAL proteins for the effect on biofilm formation, we further investigated the effect of over-production of these proteins on the surface associated motility of *A. baumannii*17978.

**Figure 7:**
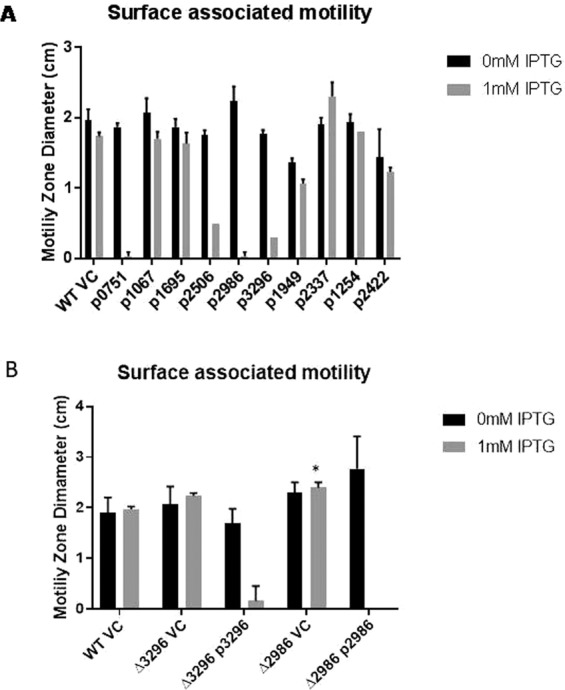
Surface associated motility of *A. baumannii* 17978. The assay with quantification of surface associated motility was performed as described in materials and methods. Error bars represent mean ± SD values of three experiments. (**A**) Motility upon the expression of individual GGDEF/EAL domain proteins from the plasmid pMMB67EH after 7 hours of incubation. (**B**) Motility upon deletion of coding sequences for *A1S_2986* and *A1S_3296*, respectively, from the genome A. baumannii. *Represents *p of* value of less than 0.05 upon student paired *t test* as compared to WT VC (Wild type vector control).

Surface associated motility assay revealed that the expression of GGDEF domain proteins A1S_0751, A1S_2506, A1S_2986 and A1S_3296 from plasmid caused distinct suppression, whereas the expression of GGDEF-EAL domain protein A1S_2337 caused a slight enhancement, of the surface associated motility of *A. baumannii* when compared to parental strain (Fig. 7A). Both these effects were observed in presence of IPTG induction that strongly suggests that the surface associated motility is regulated by c-di-GMP signalling that depends on GGDEF/EAL proteins. The derivatives expressing the rest of the GGDEF/EAL proteins did not exhibit statistically significant alterations in surface associated motility. Since the over-production of A1S_2986 exhibited the most pronounced effect on motility, the gene encoding *A1S_2986* was deleted from the genome of *A. baumannii* 17978. The capability of *A1S_2986* and *A1S_3296* mutants for surface associated motility was monitored (Fig. 7B). Both strains appeared somewhat more motile than the wild type. The trans-complementation of each mutant strain by the respective expression plasmid led to complete inhibition of motility upon induction with IPTG (Fig. 7B).* A. baumannii* does not express flagella and twitching motility is achieved by type IV pili. However, the precise mechanism of surface associated motility remains to be elucidated. The present findings that A1S_3296 caused reduced presence of pili like structures on the bacterial surface (Fig. 6C) and similarly caused inhibition of surface associated motility (Fig. 7B) may indicate that the pili structures have a role in this type of motility.

### A1S_3296 regulates biofilm formation through Csu pili dependent pathway

Our results on macro colony formation, biofilm formation and surface associated motility suggest that GGDEF domain protein A1S_3296 is a major diguanylate cyclase that positively regulates biofilm formation and negatively regulates surface associated motility in *A. baumannii*. Therefore, we further investigated the role of A1S_3296 in biofilm formation. In *A. baumannii* Csu pili and type IV pili are known to be important for biofilm formation^24,34^.

**Figure 8:**
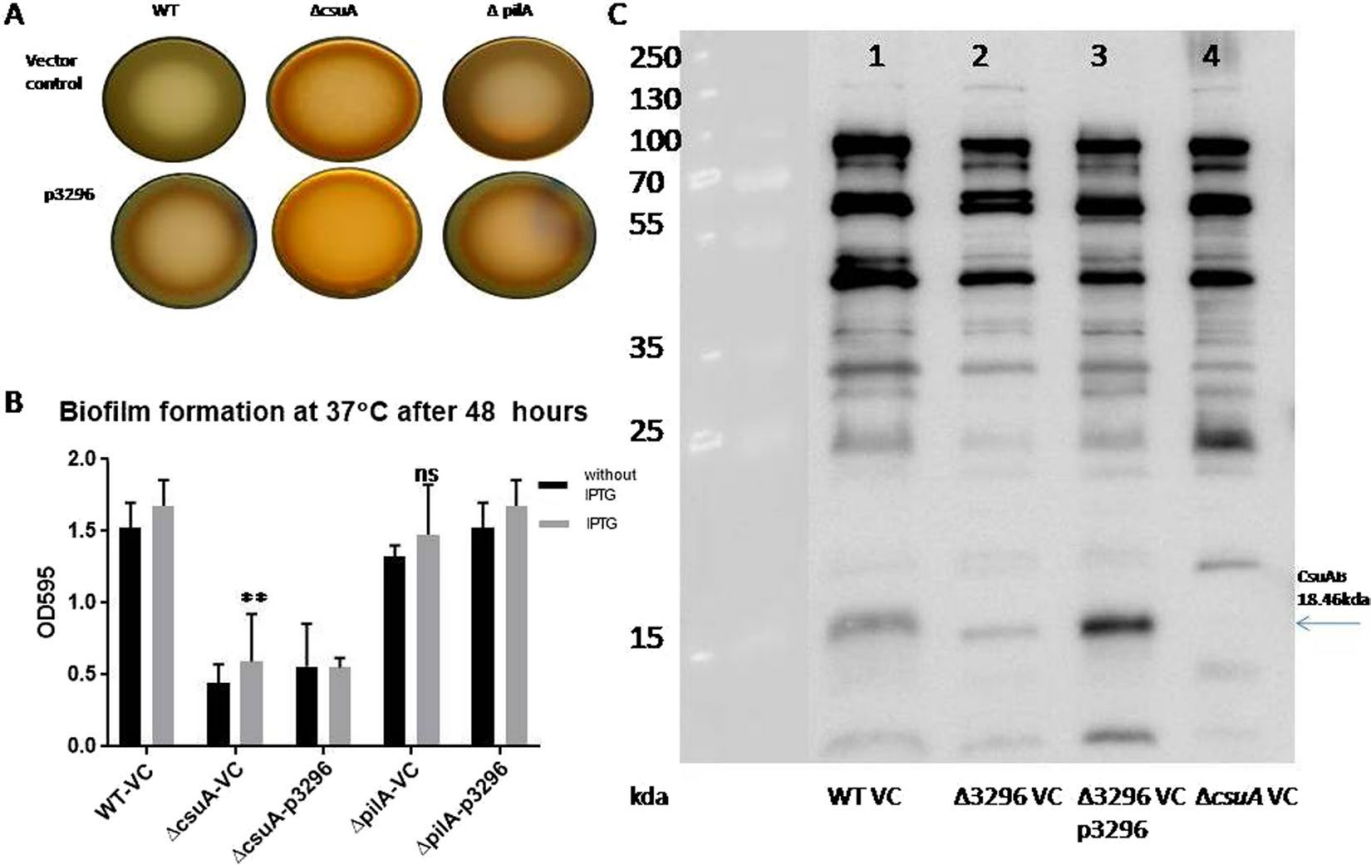
A1S_3296 promoted biofilm formation through Csu pili mediated pathway. The effect of A1S_3296 in *csuA* and *pilA* mutants regarding congo red binding in macro colonies (**A**) and biofilm formation. (**B**) Immunoblot blot to illustrate alteration in CsuAB expression in 2396 mutant as compare to wild type and complemented strain. (**C**) The strain lacking the expression of *csuABC* operon (*∆csuA*) was used as negative control for CsuAB expression.

In order to investigate the role of A1S_3296 in Csu pili and type IV pili mediated regulation of biofilm formation, we created deletion mutants of *pilA* encoding major subunit of type IV pili and *csuA* encoding subunit of Csu pili. The *csuA mutant* exhibited significant reduction in biofilm formation and pellicle formation capabilities as compare to wild type strain. Interestingly, *pilA* mutant though exhibited slight reduction in biofilm formation capability but pellicle forming capability was not reduced as compare to wild type. This finding suggests that csu pili plays major contribution in pellicle formation in *A. baumannii*. The over expression of A1S_3296 could not activate biofilm formation, pellicle formation and Congo red binding in csuA mutant (Fig. 8). Moreover, the expression of CsuAB, the major subunit of csu pili was found to be diminished in the pellicle of A1S_3296 mutant as compare to wild type. The over expression of A1S_3296 through plasmid enhanced the expression of CsuAB in *A. baumannii* (Fig. 8D). These finding suggests that A1S_3296 regulates Csu pili mediated biofilm formation.

## Discussion

The c-di-GMP signalling network of *A. baumannii* here identified as eleven GGDEF/EAL domain proteins was characterized phenotypically for enzymatic activity in the two model organisms *S. typhimurium* and *V. cholerae* where c-di-GMP signalling has been extensively studied. The rdar morphotype of *S. typhimurium* and rugose morphotype of *V. cholerae* are known to be rapidly altered by slight fluctuations of c-di-GMP levels in the cell^25,29^. Therefore, these models were here used to test c-di-GMP metabolizing capacity of *A. baumannii* GGDEF/EAL proteins. We propose here that six GGDEF domain proteins as active diguanylate cyclases and four EAL proteins as active phosphodiesterases based on their capability to alter rdarmorphotype and rugose morphotype formation.

Occurrence of GGDEF/EAL domain proteins is not enough as an argument to propose an active c-di-GMP signalling network in bacteria because of the fact that genes for enzymatically inactive GGDEF/EAL domain proteins abundantly exist in bacterial genomes. For example, c-di-GMP signalling was reported to not exist in *Staphylococcus aureus* although there is a GGDEF domain protein, GdpS, that regulates biofilm formation independent of c-di-GMP signalling^26^. Similarly, EAL proteins YcgF, YdiV in *E. coli* and STM1697 in *S. typhimurium* have been shown as enzymatically inactive proteins^19,27,28^. However, we successfully demonstrated that most of the predicted GGDEF/EAL proteins of *A. baumannii* are enzymatically active. Therefore, here we propose for the first time that an active c-di-GMP signalling system exists in *A.baumannii* and that a panel of GGDEF/EAL domain proteins consisting of A1S_2506, A1S_1949 could regulate biofilm formation in a temperature dependent manner when expressed through plasmid whereas A1S_3296 seems to regulate biofilm formation independent of temperature (Fig. 5A,B). Similarly, another panel of GGDEF/EAL proteins consisting of A1S_0751, A1S_2506, A1S_2986 and A1S_3296 (Fig. 7) could regulate surface associated motility upon over expression.

**Figure 9:**
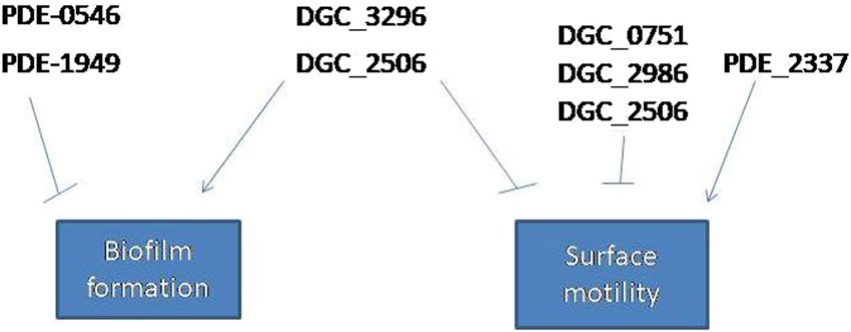
Summary of GGDEF/EAL proteins *A, baumannii* 17978 that regulate biofilm formation and surface associated motility. Arrows represent enhancement of the phenotype whereas line bars represent suppression of the phenotype.

Interestingly, sequence analysis and phenotypic analyses in model organisms suggest that six GGDEF proteins are active diguanylatecyclases. Only two of these proteins, A1S_3296 and A1S_2506,were found to be associated with regulation of biofilm formation in liquid culture. Therefore, it can be speculated that some c-di-GMP receptor(s) involved in regulating biofilm formation may be compartmentalized or signal specific and therefore not accessible to every diguanylate cyclase. The panels of GGDEF/EAL proteins and their enzymatic identities here found to regulate biofilm formation and surface associated motility are summarised in Fig. 9.

Existence of c-di-GMP in the form of dedicated pools has been indicated in many bacteria. In *S. typhimurium*, the c-di-GMP synthesized by GGDEF domain protein AdrA specifically activates cellulose synthase BcsA to synthesize cellulose fibres^29^. Similarly, a distinct panel of GGDEF/EAL proteins regulate in *S. typhimurium* the invasion into epithelial cells, IL-8 induction, its colonization of the gut in the mice model and its biofilm formation^30,31^.

GGDEF domain proteins A1S_2506, A1S_2986 and A1S_3296 promoted the expression of some extra-cellular matrix component(s), which turned reddish upon congo red binding, is a novel phenotype observed in *A. baumannii* (Fig. 4). Scanning electron microscopic imaging of macro colonies confirmed that the extracellular matrix component associated with over-expression of A1S_3296 was secreted. Previously, extracellular cellulose synthesized by *E. coli, S. typhimurium* and *G. xylinus* has been shown to bind the congo red dye^23^. However, a cellulose biosynthesis machinery does not exist in *A. baumannii*. Identification of some congo red binding matrix component from *A. baumannii* is subject to follow up studies as it may lead to development of additional convenient tools for monitoring of biofilm formation using congo red plates.

AIS_3296 appeared to be a major diguanylate cyclase regulating biofilm formation, macro colont formation and surface associated motility. However, this protein is lacked of apparent N terminal sensor domain (Fig. 1). There might be possibility that some adopter protein interacts with A1S_3296 to stimulate enzymatic activity. Actions of GGDEF/EAL proteins through protein protein interactions are now being frequently reported^32^. The molecular mechanisms of A1S_3296 mediated regulation of target processes will be an open question for follow up studies.

Biofilm formation is a strategic trait of many bacteria adopted to survive in harsh environments. The c-di-GMP molecule is one of the central regulatory components of biofilm formation and therefore identification of inhibitors of A1S_3296 might lead to development of disinfectant to prevent *A. baumannii* biofilms and colonization. Further elucidation of specific c-di-GMP receptors regulating biofilm formation and motility will be interesting topics of follow up studies.

## Methods

### Bacterial strains and growth conditions

All the strains used in this study are listed in Table S1. Strains were stored in 15% glycerol solution in LB broth at −80 °C. The growth of strains was maintained in LB broth or on LB-agar plates. In order to maintain pMMB67EH, 100 µg/ml carbenicillin was supplemented to the media. 1 mM Isopropyl β-D-1-thiogalactopyranoside (IPTG) was used to induce expression of genes through pMMB67EH.

### Plasmids construction

All the plasmids are listed in Table S2. In total, genes for eleven GGDEF/EAL domain proteins of *A. baumannii* 17978 were cloned into broad host vector pMMB67EH^33^ as C-terminal 6xHis fusion constructs. DNA sequences including open reading frames along with ribosomal binding region of individual genes were amplified from template DNA isolated from *A. baumannii* 17978 using Phusion Polymerase (Thermo scientific). Resulting PCR products were digested with high fidelity endonucleases (Thermo scientific) in accordance with the harboured restriction sites. A combination of *Sac*1/*Bam*H1 endonucleases was used to digest *A1S_0546*; a combination of *Sac*1/*Xba*1 to digest *A1S_0751, A1S_2986*, *A1S_3296* and A1S_2422*;* a combination of *Kpn*1/*Xba*1 to digest *A1S_1695*, a combination of *Eco*R1/*Sac*1 to digest *A1S_1949*; a combination of *Bam*H1/*Sal*1 to digest *A1S_2337* and a combination of *Eco*R1/*Bam*H1 was used to digest *A1S_2506*. Digestion products were purified with gene clean kit (Thermo scientific) and ligated with the pMMB67EH vector digested with same combination of enzymes.

For each construct, the ligation mixture was introduced into chemo competent *Escherichia coli Dh5α* by heat shock at 42 °C for 1 minute with subsequent growth in 2x YT broth at 37 °C for 1 hour. The transformants of *E. coli* were selected on agar plates containing carbenicillin (100 μg/ml) and confirmed by PCR, using forward primer upstream and reverse primer downstream of multiple cloning sites. The integrity of the construct was confirmed by DNA sequencing of the inserted genes; performed by Eurofins GATC Biotech, Germany.

Plasmids were introduced into *V.cholerae* C6706*luxO*^*C*^*, A. baumannii* 17978 and *S. typhimurium* SR11 by electroporation at 1,8 mV voltage and 25 Ω resistance. Electro-competent cells were prepared by washing the cells grown into logarithmic phase with 10% ice-cold glycerol three times. Since *V. cholera* is sensitive to low osmolarity, washing solution contained additional 1 mM MgCl_2_. For electroporation_,_ five micro liters of plasmid preparations were added to 200 micro liters of competent cells. The electroporation cuvettes of 2 mm gap (Cell Projects) were used. Upon electric shock at 1.8 mV voltage and 25 Ω resistance, cells were incubated at 37 °C for 1 hour in 2x YT broth. The transformants that acquired pMMB67EH were selected on agar plates containing carbenicillin (100 μg/ml).

### Creation of mutant strains of *A. baumannii* 17978

Chromosomal mutants of *A. baumannii* 17978 for *A1S_3296, A1S_2986* and *pilA* were generated as a one-step gene replacement by homologous recombination^24^. The entire open reading frames except 100 nucleotides at the beginning and at the end of the gene were replaced by a kanamycin resistance marker. The kanamycin gene, along with sequences corresponding to the target gene such that there were homologous overhangs of 100 bps, was PCR‐amplified from pKD4 and introduced by electroporation into *A. baumannii* carrying pAT02. pAT02 is a derivative of pMMB67EH where recombinase RecAB is inserted. Oligonucleotide primers used are described in Table S3. Prior to electroporation, *A. baumannii* carrying pAT02 was grown to log phase in LB broth containing carbenicillin (100 μg/ml) and IPTG (2 mM). After 3 washes with ice-cold 10% glycerol and subsequent 1000-fold concentration of the bacterial sample, 100 µl of bacterial suspension (~10^10^ bacteria) were mixed with 5 µg of recombineering PCR product and electroporated in a 2-mm cuvette at 1.8 kV. After outgrowth in 4 ml rich medium containing 2 mM IPTG, the bacteria were pelleted, plated on LB-agar with 7.5 µg/ml kanamycin, and incubated overnight at 37 °C. All constructed mutants were verified by PCR with control primers matching to sequences in the genes flanking the deleted open reading frame.

### Scanning electron microscopy

For Scanning electron microscopy, five microliters of bacterial suspension in PBS (OD_600_ of 1) from an overnight plate culture were spotted onto LB agar plates. The plates were incubated at 37 °C for 24 hours. Pieces of agar containing bacterial colonies were removed and fixed overnight at 4 °C with 2.5% glutaraldehyde in 0.1 M sodium cacodylate, dehydrated in graded series of ethanol, critical point dried and coated with 5 nm gold/palladium. The bacterial cells morphology were analyzed by field-emission scanning electron microscope (Carl Zeiss Merlin FESEM) using secondary electron detectors at accelerating voltage of 4 kV and probe current of 50–100 *p*A.

### Macro colony formation assay

Development of colony formation on agar plates is a multi-cellular behavior of bacterial isolates. The red dry and rough (rdar) morphotype of *S. typhimurium* and rugose morphotype of *V. cholera* on agar plates supplemented with congo red dye are typical examples of biofilm formation as macro colonies. The rdar morphotype of *S. typhimurium*, rugose morphotype of *V. cholerae* and colony formation of *A. baumannii* were observed on agar plates (LB without salt) supplemented with 0.02 μg/ml congo red (Sigma Aldrich) and 0.01 μg/ml Brilliant blue G (Sigma Aldrich). Five microliters of bacterial suspension in PBS (OD_600_ of 1) from an overnight plate culture were spotted onto Congo Red plates supplemented with 100 µg/ml carbencillin and 1 mM IPTG. The plates were incubated at 30 °C or 37 °C. Macroscopic and stereomicroscopic images were taken after 48 and/or 72 hours. Congo Red plates with bacteria after incubation without IPTG were used as a non-inducing control of investigated genes in each experiment.

### Biofilm formation assay

Biofilm formation assay was performed in sterilized 96 well microtiter plates (Nunc^TM^ Cell culture treated, Thermo scientific). PBS suspension of bacterial cells grown overnight on LB agar plates was prepared to OD_600_ = 1. From this suspension, twenty micro liters were added to each well of 96 well plates containing 180 micro liters of LB broth supplemented with 100 μg/ml carbenicillin with or without 1 mM IPTG. Plates were incubated in a moist chamber at 30 °C or 37 °C for 24 hours. Subsequently, the liquid contents were discarded and the plates washed gently with water. Bacterial cells attached to walls, or at the base, of wells in the form of biofilm were stained with 1% crystal violet for 30 minutes. Excessive crystal violet was removed by washing three times with water. Cells stained with crystal violet were dissolved in 5% acetic acid solution. The intensity of the crystal violet colour represents the abundance of biofilm and it was measured as optical density with a 595 nm filter. Data were subjected for statistical analysis using Graph Pad Prism software.

### Surface associated motility assay

Surface associated motility assay was performed in petri plates containing soft agarose medium as described previously^10^. Soft agarose media consists of tryptone (5 g/l), agarose (5 g/l) and sodium chloride (2,5 g/l). For motility assay, five microliters of bacterial suspension in PBS (OD_600_ of 1) from an overnight plate culture were spotted onto soft agarose plats supplemented with 100 µg/ml carbenicillin with or without 1 mM IPTG. The plates were incubated at 37 °C for 7 hours. Diameter of motility zone was recorded and analyzed using Graph Pad prism software.

### Western blotting

Western blotting for CsuAB was performed as described previously with slight modification^34^ from the bioflm mass grown in pellicle. Briefly,vernight bacterial cultures were diluted 1:100 in 5 mL of LB broth and grown stagnant in polystyrene tubes for 48 hours hours at 25 °C without shaking in the dark. Cells in air-liquid interphase formed pellicle. Cells grown in pellicle were mixed with Laemmli buffer and boiled for ten minutes. The proteins were separated by electrophoresis in 18% SDS polyacrylamide gels and transferred onto an immunoblot polyvinylidene difluoride membrane (Bio-Rad Laboratories) in Bio-Rad A-buffer (25 mM Tris, pH 8.3, 192 mM glycine, with 20% methanol and 0.1% SDS) at 100 V for 1 h. Membrane was blocked with 5% skim milk in PBS/Tween, incubated with primary anti-CsuA/B rabbit polyclonal antibody (Innovagen AB), followed by incubation with secondary HRP-conjugated anti-rabbit goat antibody (AgriSera AB, Sweden). For A1S_3296-6xHis detection, *S. typhimurium* SR11 strains were grown in LB without salt agar plates at 30 °C for 24 hours. Cells were resuspended in Laemmli buffer and boiled for ten minutes. The proteins were separated by electrophoresis in 10% SDS polyacrylamide gels and transferred onto an immunoblot polyvinylidene difluoride membrane (Bio-Rad Laboratories) in Bio-Rad A-buffer (25 mM Tris, pH 8.3, 192 mM glycine, with 20% methanol and 0.1% SDS) at 100 V for 1 h. Membrane was blocked with 5% skim milk in PBS/Tween, incubated with monoclonal anti- polyhistidine antibody (H1029, Merck) used as 1:5000 dilution followed by incubation with secondary HRP-conjugated anti-mouse antibody (Dako, Denmark).

## Supplementary information

Supplementary information
