# Supplementary material for: A Cyclic-di-GMP signalling network regulates biofilm formation and surface associated motility of Acinetobacter baumannii 17978
Source: Sci Rep. 2020 Feb 6;10:1991. doi: 10.1038/s41598-020-58522-5 (PMC7005169; doi:10.1038/s41598-020-58522-5)
Supplement: Supplementary file 1 — Supplementary information [file 41598_2020_58522_MOESM1_ESM.docx]

Supplementary information

A Cyclic-di-GMP signalling network regulates biofilm formation and surface associated motility of Acinetobacter baumannii 17978

^1,2^ Irfan Ahmad, ^1^Evelina Nygren, ^2^Fizza Khalid, ^1^Si Lhyam Myint and ^1^Bernt Eric Uhlin

^1^The Laboratory for Molecular Infection Medicine Sweden (MIMS) and the Department of Molecular Biology, Umea University, Umea, Sweden

^2^Institute of Biomedical and Allied Health Sciences, University of Health Sciences, Lahore, Pakistan

**Figure S1**

**
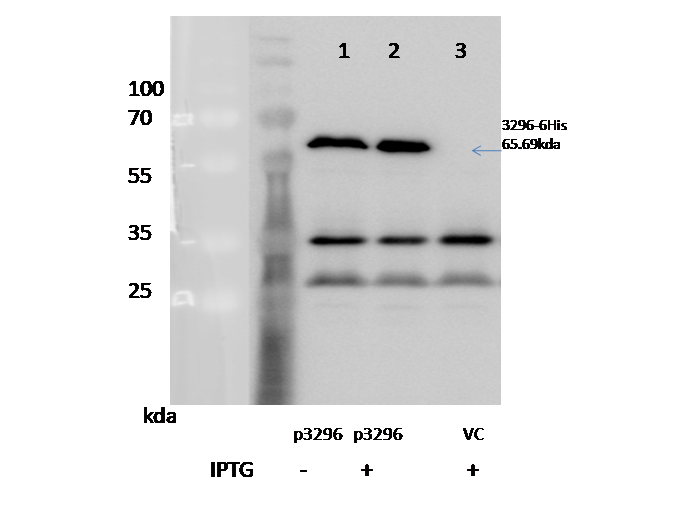
**

**Figure S2: Immunoblot illustrating the expression of A1S_3296-6His through plasmid pMBB67EH in the presence and absence of inducer IPTG.** 1&2: *A. baumannii* 17978 containing pMBB67:A1S_3296, 3: *A. baumannii* 17978 containing pMBB67 as a vector control (VC)

**Table S1. Bacterial strains used in the study**

| Strain | Genotype | Reference |
| --- | --- | --- |
| *Escherichia coli*DH5α | wild type | ATCC |
| *Salmonella typhimurium* SR11 | wild type | ATCC |
| *Vibrio cholerae*C6706*luxO^C^* |  | (Vance et al., 2003) |
| *Acinetobacter baumannii*ATCC 17978 | wild type | ATCC |
| *Δ3296:Km* | *Acinetobacter baumannii*ATCC 17978where *A1S_3296* is replaced by Km resistance gene | This study |
| *Δ2986:Km* | *Acinetobacter baumannii*ATCC 17978where *A1S_2986* is replaced by Km resistance gene | This study |
| *ΔpilA:Km* | *Acinetobacter baumannii*ATCC 17978where  *pilA* is replaced by Km resistance gene | This study |
| *ΔcsuA:Km* | *Acinetobacter baumannii*ATCC 17978where *csuA* is replaced by Km resistance gene | This study |

**Table S2. Plasmids used in the study**

| Plasmid | Genotype | Reference |
| --- | --- | --- |
| pMMB67EH | Broad host range | (Fürste et al., 1986) |
| p0751 | pMMB67EH clone with locus A1S­_0751 | This study |
| p1067 | pMMB67EH clone with locus A1S­_1067 | This study |
| p2986 | pMMB67EH clone with locus A1S­_2986 | This study |
| p3296 | pMMB67EH clone with locus A1S­_3296 | This study |
| p1695 | pMMB67EH clone with locus A1S­_1695 | This study |
| p2506 | pMMB67EH clone with locus A1S­_2506 | This study |
| p0546 | pMMB67EH clone with locus A1S­_0546 | This study |
| p1949 | pMMB67EH clone with locus A1S­_1949 | This study |
| p2337 | pMMB67EH clone with locus A1S­_2337 | This study |
| p1254 | pMMB67EH clone with locus A1S­_1254 | This study |
| p2422 | pMMB67EH clone with locus A1S­_2422 | This study |
| pPilA | pMMB67EH clone with locus *pilA* | This study |

**Table S3.Primers used in the study**

| Primer | 5`-3` Squence |
| --- | --- |
| Cloning Primers |  |
| 0751_F | TCGCGAGCTCTGTAGGAACAAATTTATGATTGGTAGTTTTTGG |
| 0751_R | AAAGTCTAGATTAGTGATGGTGATGGTGATGCAAAGAAATAAAAACTCTATTACGC |
| 1067_F | GAAGGATCCTGTAGGAATCGACTAGTGGCGAATAGG |
| 1067_R | TTCGTCGACTTAGTGATGGTGATGGTGATGGGCAATTTCAGCGACTTGTA |
| 1695­­_F | TCGCGGTACCGAGAGCATTAATTATGAAGTTGCAAGG |
| 1695_R | AAAGTCTAGATTAGTGATGGTGATGGTGATGAGTTAGATCTACAATTTGTTCTTC |
| 2506_F | CCTGGAATTCTGTAGGAAGAAACGCATGGAAACTTTAGATTCTTC |
| 2506_R | CGCAGGATCCTTAGTGATGGTGATGGTGATGTGGTAGAAGTAATTTCTTG |
| 2986_F | TCGCGAGCTCTGTAGGAATTTTACGATGGAAACTTATGCAAAAC |
| 2986_R | AAAGTCTAGATTAGTGATGGTGATGGTGATGATTCTCTGGTTTATAAATAAACC |
| 3296_F | TCGCGAGCTCTGTAGGAAGCTAGAAATGTCAGGATTACGTTCA |
| 3296_R | AAAGTCTAGATTAGTGATGGTGATGGTGATGTCCCTCTACTACATTTCGTCC |
| 1949_F | CCTGGAATTCTGTAGGAAGCATGTTATGACTTCAATGT |
| 1949_R | TTCGTCGACTTAGTGATGGTGATGGTGATGATTCATAGAATAGTGACTTTCTAGATA |
| 2337_F | GAAGGATCCAGGATAGAAAAATCTTATGTCTGG |
| 2337 – Rev | TTCGTCGACTTAGTGATGGTGATGGTGATGAATATGCGTATTTGTTTGTAGATATTG |
| 1254_F | GAAGGTACCTGTAGGAATCTTTATATGGATATCTGTTTC |
| 1254_R | TTCGTCGACTTAGTGATGGTGATGGTGATGTTCTTTATCGATCTTGATGGGCAAT |
| 2422_F | TCGCGAGCTCTGTAGGAAAAGATTTATGGGAAGCATTAAAGTGAG |
| 2422_R | AAAGTCTAGATTAGTGATGGTGATGGTGATGTTGGTCTTGTACGTCTGTTAAGC |
| 0546_F | CCTGGAGCTCTGTAGGAAGTCCAATATGGGTCATGTTGATTACGA |
|  |  |
| 0546_Rev | CGCAGGATCCTTAGTGATGGTGATGGTGATGAACAAAATGAGCACCTTGCGACTGAAT |
| pilA_F | CGTATCTAGAGGGAAAAAGGCTATGAATGCAC |
| pilA_R | CGTCGAAGCTTTTAATGGTGATGGTGATGGTGAATTATTGTACAGCCTTTTGGAGC |
| Sequencing Primers |  |
| pMMB67EH - forward | CACTGCATAATTCGTGTCGCTC |
| pMMB67EH – reverse | CTGTATCAGGCTGAAAATCTTCTC |
| 0546_Seq | CCAATATCAGGCTGAACATGAC |
| 2337_1 | CAAATGCCTTGTAGCACTAA |
| 2337_2 | ACCTTCCTAACCGCTTTTACT |
| 2337_3 | CACATATCATCTTGATGGTTC |
| Gene deletion Primers |  |
| pilA-ko-1f | ATGAATGCACAAAAAGGTTTTACATTAATTGAACTCATGATCGTGGTTGCCATTATCGGTATTTTGGCAGCAATTGCGATTCCGCAATATCAGAAGATTGTGTAGGCTGGAGCTGCTTCC |
| pilA-ko-1r | TTAAATTATTGTACAGCCTTTTGGAGCAATTACTGAAGAAATATCTGTTGTTCCTTTAGTAATAGAACATGTCCAGCCACCCACATTTGCATATGAATATCCTCCTTAGTTCCTATTCCG |
| pilA-control-f | GCAATACCAGAAAGCTGTAGTTAC |
| pilA-control-rev | CTTAACCCTGCTGCAAAGGCA |
| A1S_2986_Ko_F | ATGGAAACTTATGCAAAACAAAATTTGCAATTACTTAGCCATACTCTTCTTGAGCGTATTCAACCTGCCGTTGTGTTTAACGATAAGATCACGATCCCGGAATTGCCAGCTGGG |
| A1S_2986_Ko_R | TTAATTCTCTGGTTTATAAATAAACCAATGTTGCTCAGAAGTCTTAGCCTTATACATCGCTTGATCTGCTTGCATAATAAAATCTTCTGGATTCAGAAGAACTCGTCAAG |
| A1S_2986_Control_F | TTGGCTAAGAACGCGGAGTAA |
| A1S_2986_Control_R | GCACAGCAATGCAATAAAGG |
| A1S_3296_ko_F | ATGTCAGGATTACGTTCAGAACATATCAGTAATCGACTGTTTTTCTTCATGTTGGTCATTATTTTGTCCTTACTGTTTATGGCGGTTCCGCTTATTCCGGAATTGCCAGCTGGG |
| A1S_3296_ko_R | TTATCCCTCTACTACATTTCGTCCCCGAGCTTTAGCTCTTAAGAGTGAACGATCAGCGTTCTCTAATAACTCCATCCAGCTTGTCGCTCCATTCAGAAGAACTCGTCAAG |
| A1S_3296_control_F | ACAGCAGTGGCTAAACCAAG |
| A1S_3296_control_R | GCTTCTTGAAACCGATGTTGA |
| csuA_ko_F | ATGATATTCAATCGTGGTTCGGCATTTATAATTTCTTATTTTTTAATTTCTTTAGTAAATGCGGGTGAAATTGGAGCTAAATTAACTAGTCAAATTGAATCCGGAATTGCCAGCTGGG |
| CsuA_ko_R | TTAAAACTCAATCGTAATTGGTACTATATCTTTATATTCACCCTTAGATATACGACTACCATCATGGGTTGCTTGACCAAAAATATCGATATTCTTTTTATTCAGAAGAACTCGTCAAG |
| CsuA_Control_F | GGCGATTATAAAGATACTC |
| CsuA_Control_R | GAATTGAGCTGAGGATCT |
